# Exploring the Social Determinants of Mental Health by Race and Ethnicity in Army Wives

**DOI:** 10.1007/s40615-023-01551-3

**Published:** 2023-03-23

**Authors:** Jessica Dodge, Kathrine Sullivan, Edward Miech, Adriane Clomax, Lyndon Riviere, Carl Castro

**Affiliations:** 1 Center for Clinical Management Research, Health Services Research and Development, VA Ann Arbor Healthcare System, Ann Arbor, MI USA; 2 Silver School of Social Work, New York University, 1 Washington Square North, New York, NY 10003 USA; 3 Regenstrief Institute, Center for Health Services Research, 1101 W 10th Street, Indianapolis, IN 46202 USA; 4 Center for Innovation and Research on Veterans and Military Families, Suzanne Dworak-Peck School of Social Work, 669 West 34th Street, Suite 201D, Los Angeles, CA 90089 USA; 5 Walter Reed Army Institute of Research, 503 Robert Grant Ave., Silver Spring, MD 20910 USA

**Keywords:** Social Determinants of Health, Mental health, Military spouses, Army, Coincidence analysis, Qualitative comparative analysis

## Abstract

**Objective:**

To explore the social determinants of mental health (SDoMH) by race/ethnicity in a sample with equal access to healthcare. Using an adaptation of the World Health Organization’s SDoMH Framework, this secondary analysis examines the socio-economic factors that make up the SDoMH by race/ethnicity.

**Method:**

This paper employed configurational comparative methods (CCMs) to analyze various racial/ethnic subsets from quantitative survey data from (*N* = 327) active-duty Army wives. Data was collected in 2012 by Walter Reed Army Institute of Research.

**Results:**

Initial exploratory analysis revealed the highest-scoring factors for each racial/ethnic subgroup: *non-Hispanic Black*: employment and a history of adverse childhood events (ACEs); *Hispanic*: living off post and a recent childbirth; *junior enlisted non-Hispanic White*: high work-family conflict and ACEs; *non-Hispanic other race*: high work-family conflict and not having a military history. Final analysis showed four models consistently explained clinically significant depression symptoms and four models consistently explained the absence of clinical depression symptoms, providing a solution for each racial/ethnic minority group (non-Hispanic Black, Hispanic, junior enlisted non-Hispanic White, and non-Hispanic other).

**Discussion:**

These findings highlight that Army wives are not a monolithic group, despite their collective exposure to military-specific stressors. These findings also highlight the potential for applying configurational approaches to gain new insights into mental health outcomes for social science and clinical researchers.

**Supplementary Information:**

The online version contains supplementary material available at 10.1007/s40615-023-01551-3.

## Introduction

Active-duty military spouses experience unique stressors, including prolonged separations from their partner during deployments or training and frequent relocation [1, 2]. While most military wives cope successfully with these stressors, some evidence suggests racial/ethnic minority spouses may be at greater risk for adverse mental health outcomes [3–5]. Though minimal research has focused on this population [6], the military is a unique environment in which to study health outcomes for racial/ethnic minority populations, as the availability of universal healthcare coverage removes a significant barrier to care [7, 8]. Recent research has shown that baseline health insurance coverage actually protects Blacks more than Whites in developing chronic medical conditions over time [9]. Therefore, the military context offers a unique opportunity to understand health disparities, holding constant a key determinant of health. Using an adaptation of the World Health Organization’s (WHO) Social Determinants of Health (SDoH) framework, this paper examines the complex interrelationship between various environmental factors on mental health among different racial/ethnic groups of military wives.

## Mental Health Among Minority Spouses

In the US military, every service member and their family is provided healthcare through TRICARE, including mental healthcare [8]. Prior studies have suggested that racial/ethnic disparities in health outcomes may be driven by differences in insurance coverage and associated access to care [10, 11]. Widespread access to health insurance coverage could explain why several large-scale studies have not found racial/ethnic minority differences in mental health outcomes among military spouses [3, 12, 13]. However, other studies examining military spouses’ mental health have found increased risk for spouses from minority racial/ethnic groups [4, 14]. Further complicating this picture, some research on spousal mental health has failed to consider race/ethnicity entirely (e.g. [15, 16]). More research is needed to understand the relationship between race/ethnicity and military spouse mental health, which may inform efforts to address health disparities broadly.

## Conceptual Framework

**Fig. 1:**
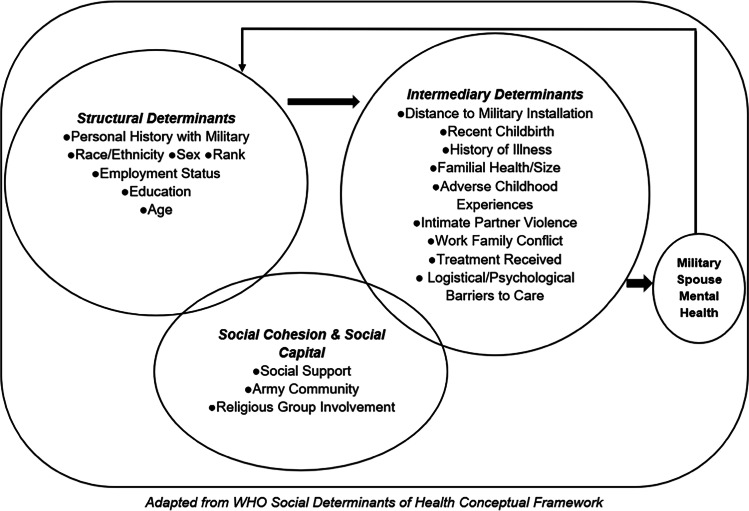
Social determinants of mental health conceptual framework for civilian military spouses

The SDoH have been conceptualized as a broad set of conditions that impact overall health [7]. This framework highlights (1) structural determinants, including demographic characteristics like race/ethnicity, sex, socio-economic status, educational attainment, and age; (2) social cohesion and capital, including social supports that can prevent or exacerbate illness; and (3) intermediary determinants, including access to resources, psychosocial factors, physical factors, and healthcare coverage. Here, this model has been adapted to focus on social determinants of *mental* health (SDoMH) and includes features unique to military families; the adapted framework specifically addresses the relationship between racial/ethnic minority status and mental health among civilian military spouses (see Fig. 1).

### Structural Determinants

Structural determinants are factors that determine social hierarchies which impact an individual’s mental health trajectory, including socio-economic position or cultural/societal values [7]. For military wives, socio-economic position may include race/ethnicity, their partner’s rank, and employment [1,3,13]. While military spouses are provided equal access to healthcare, they still operate within US society in which they are likely to be exposed to structural racism that can negatively impact mental health [17, 18]. Specifically, prior research suggests spouses who are a racial/ethnic minority, unemployed, married to a junior ranking service member, or have less education are at greater risk for stress and depression [3,13], Further, in this population, both personal history with the military and mental health stigma could create or contribute to cultural experiences or values which may impact spouse mental health. For example, spouses who have previously served in the military are at greater risk for depression, potentially due to prolonged exposure to military stressors [3]. Finally, mental health stigma is prevalent in military culture which could affect mental health through its impact on treatment seeking [19, 20].

### Social Cohesion and Capital Determinants

Social cohesion and capital are conceptualized as the “extension of social relationships and the norms of reciprocity, influencing health by way of the social support mechanisms that these relationships provide to those who participate in them.” ([7], p. 41). The Silva et al. [21] review of the social determinants of mental health literature highlights the importance of safe social connections in preventing mental illness initiation and continuation. In the context of the military, social connections may include the presence of social supports and sense of community. Military spouses with access to formal and informal social support, including religious groups, community, friends, or family, had lower levels of anxiety during military separations [22–24]. Additionally, a greater sense of community connection to the military may protect against mental health symptoms [25]. Research also documents racial differences in the protective nature of perceived social support on health outcomes overtime [26].

### Intermediary Determinants

Intermediary determinants directly influence an individual’s mental health and include material circumstances, physical factors, psychosocial factors, and their interaction with the health system. For military wives, material circumstances may include living on or off a military installation. Healthcare, subsidized food, gas, and financial assistance are all available on military installations; living off base could impact access to these resources. Among military spouses, recent childbirth and prior history of illness are physical factors that have been associated with adverse health [27].

Psychosocial factors associated with mental health among military spouses include the health/mental health of the service member, family size, the health of family members, work-family conflict, and history or presence of violence [13,28–31]. Poor health among service members can create strain for spouses who may experience additional caregiver burden, which can increase depression [32]. Military spouses with four or more children may be at greater risk for stress and depression [3,13]. The military environment lends itself to work-family conflict due to heavy workloads for service members, and prolonged periods away from home, which may have consequences for spousal mental health [29,33].

The presence of both current and past violence are important psychosocial factors that have been known to contribute to adverse mental health [30, 31]. ACEs encompass aspects of the home environment and have been linked to negative mental health trajectories, including adult depression, for military and civilian populations [4,30]. ACEs are an important psychosocial factor, as children do not experience ACEs equally across racial/ethnic categories. For example, in a nationally representative sample of children, 61% of non-Hispanic Black children and 51% of Hispanic children experienced at least one ACE compared to 40% of non-Hispanic White children and 23% of non-Hispanic Asian children [34]. Lastly, intimate partner violence (IPV) is a known risk factor for adverse mental health both within and outside the military (see [31] for review).

Despite access to mental healthcare through TRICARE insurance [8], spouses may still experience barriers to care which may inhibit pursuit of mental health treatment, such as not knowing what services are available to them, not having the time to access them, or not being able to find a clinician they trust [35, 36]. Furthermore, spouses may experience psychological barriers including stigma surrounding mental healthcare, attitudes which are more prevalent among military wives than the general population [35]. Recent research shows that racial and ethnic minority military spouses are less likely to report logistical barriers and internalized mental health stigma compared to non-Hispanic White individuals [36].

## The Current Study

Guided by the SDoMH framework, this analysis examines how structural, social cohesion and capital, and intermediary determinants explain depression among Army wives. This study applies configurational comparative methods (CCMs), an approach well-suited to explore a complex outcome like depression among racial/ethnic minorities. CCMs systematically identify specific combinations of conditions that account for an outcome of interest and are suited to analyses with small racial and ethnic subsamples.

CCMs represent a set-theoretic analytic approach that uses Boolean algebra to evaluate how bundles of conditions yield an outcome of interest [37, 38]. In CCMs, the outcome relies on configurational patterns that identify necessity (conditions always present when the outcome is present, but alone do not guarantee the outcome) and sufficiency (conditions co-occur with outcome). These methodologies do not have the same sample size requirements as correlational methodologies [39]. CCMs identify how multiple conditions work together in configurations that operate jointly and allow for modeling equifinality, when multiple paths lead to an outcome, and conjunctivity, when a condition may only be relevant to an outcome if it is paired with another condition [37,40]. For example, CCMs have been recently used to assess pathways of help-seeking behaviors for Black male trauma survivors [41]. The ability of CCMs to detect conjunctivity and equifinality makes this approach ideal to understand the interconnected impact of Army wives’ structural, social cohesion and capital, and intermediary determinants on their mental health. One exploratory question guided the analysis: Which conditions across the three determinant categories combined to produce (or *not* produce) clinically significant depression symptoms for Army wives by race and/or ethnicity?

### Methods

#### Data and Participants

Data initially collected in 2012 by Walter Reed Army Institute of Research (WRAIR) were used for this secondary analysis. Survey data were collected from the spouses of one military unit approximately 16 months after this unit returned from Afghanistan. In-person and online surveys were administered to participants in the continental USA (see [25, 42], for more details about recruitment methods). All initial procedures and secondary analyses were reviewed by WRAIR’s Institutional Review Board. Each survey took between 30 and 45 min to complete. Twenty-three percent of spouses responded to recruitment, and 98% agreed to participate and provided informed consent (*N* = 343). The majority (74.2%) completed a web-based version of the survey, and the remainder completed a paper version. Due to low numbers, nine male spouses along with seven female spouses who indicated they were in active-duty service as their current employment were dropped for a final analytic sample of 327 female spouses. The majority of spouses were White (74.7%), unemployed (60.3%), with at least one child (63.8%). About half had some college/associates degree (49.8%) and the largest group reported their partner held a rank of E5–E9 (43.9%; see Table 1).

**Table 1:** Demographic characteristics of whole sample and each race/ethnicity subsample

Category	Characteristic	Whole sample *N* (%) / M (SD) range	NH White *N* (%) / M (SD) range	Junior enlisted NH White *N* (%) / M (SD) Range	NH Black *N* (%) / M (SD) range	Hispanic *N* (%) / M (SD) range	NH other race *N* (%) / M (SD) range
Race/Ethnicity	White	245 (75%)	245 (100%)	87 (100%)	-	-	-
	Black	22 (7%)	-	-	22 (100%)	-	-
	Hispanic	34 (10%)	-	-	-	34 (100%)	-
	Other	25 (8%)	-	-	-	-	25 (100%)
Rank	E1−E4	112 (35%)	87 (36%)	87 (100%)	3 (14%)	14 (41%)	8 (32%)
	E5−E9	142 (44%)	94 (39%)	-	17 (77%)	15 (44%)	16 (64%)
	Officer	71 (22%)	63 (26%)	-	2 (9%)	5 (15%)	1 (4%)
Employment Status	Employed	96 (30%)	73 (30%)	24 (28%)	12 (55%)	4 (12%)	7 (29%)
	Unemployed Looking For Work	71 (22%)	48 (20%)	16 (18%)	7 (32%)	12 (35%)	4 (17%)
	Unemployed Not Looking For Work	125 (39%)	99 (40%)	37 (43%)	2 (9%)	13 (38%)	11 (46%)
	Other	33 (10%)	25 (10%)	10 (12%)	1 (5%)	5 (15%)	2 (8%)
Education	Less than Bachelors	216 (67%)	158 (65%)	76 (88%)	13 (59%)	18 (82%)	17 (68%)
	Bachelor’s Degree or More	109 (34%)	86 (35%)	10 (12%)	9 (41%)	6 (18%)	8 (32%)
Age	18−29 years old	169 (52%)	133 (54%)	59 (68%)	5 (23%)	21 (62%)	10 (40%)
	30 + years old	157 (48%)	112 (46%)	28 (32%)	17 (77%)	13 (38%)	15 (60%)
Personal History with Military	No	223 (71%)	169 (71%)	64 (76%)	14 (64%)	24 (73%)	16 (80%)
	Yes	91 (29%)	70 (29%)	20 (24%)	8 (36%)	9 (27%)	4 (20%)
Social Support	Continuous Scale	11.1 (3.4) 3−15	11.2 (3.5) 3−15	11 (3.3) 3−15	10.6 (3.6) 3−15	10.2 (4.0) 3−15	11.3 (3.3) 6−15
Army Community	Continuous Scale	13.6 (3.7) 4−20	13.7 (3.8) 4−20	13.2 (3.9) 4−20	12.7 (2.9) 8−18	13.6 (3.3) 4−19	13.4 (3.4) 5−20
Religious Group Involvement	No	181 (56%)	136 (56%)	61 (71%)	10 (46%)	19 (56%)	16 (64%)
	Yes	142 (44%)	106 (44%)	25 (29%)	12 (55%)	15 (44%)	9 (36%)
Clinically Significant IPV	No	262 (85%)	203 (88%)	67 (82%)	13 (62%)	26 (79%)	20 (83%)
	Yes	48 (16%)	29 (13%)	15 (18%)	8 (38%)	7 (21%)	4 (17%)
History of any ACEs	No	128 (40%)	102 (43%)	36 (42%)	9 (45%)	8 (24%)	9 (36%)
	Yes	189 (60%)	137 (57%)	50 (58%)	11 (55%)	25 (76%)	16 (64%)
Distance to Military Installation	Live on Post	125 (39%)	91 (38%)	32 (37%)	8 (36%)	16 (49%)	10 (40%)
	Live Off Post	198 (61%)	152 (63%)	54 (63%)	14 (64%)	17 (52%)	15 (60%)
Childbirth in past year	No	251 (78%)	188 (77%)	71 (82%)	18 (82%)	27 (79%)	18 (78%)
	Yes	73 (23%)	57 (23%)	16 (18%)	4 (18%)	7 (21%)	5 (22%)
Injury/Illness in past year	No	260 (80%)	193 (79%)	66 (76%)	17 (77%)	28 (82%)	22 (92%)
	Yes	65 (20%)	52 (21%)	21 (24%)	5 (23%)	6 (18%)	2 (8%)
Does Service Member need Mental Health Treatment?	No	265 (82%)	196 (81%)	71 (84%)	15 (68%)	31 (91%)	23 (92%)
	Yes	58 (18%)	46 (19%)	14 (17%)	7 (32%)	3 (9%)	2 (8%)
Number of Children	Continuous Scale	1.2 (1.3) 0−5	.55 (.87) 0−5	.40 (6.5) 0−3	.38 (6.2) 0−2	.52 (.75) 0−2	.2 (.56) 0−2
Work-Family Conflict	Continuous Scale	22.1 (7.7) 5−35	22.5 (7.4) 5−35	20.3 (7.8) 5−35	22.1 (7.7) 10−34	20.9 (9.4) 5−35	20.1 (8.3) 5−25
Current MH Tx received	No	299 (92%)	223 (91%)	76 (87%)	21 (96%)	30 (91%)	25 (100%)
	Yes	26 (8%)	22 (9%)	11 (13%)	1 (5%)	3 (9%)	0
Logistical barriers to care	Continuous Scale	8.1 (3.1) 4−18	8.1 (3.1) 4−18	8.0 (3.5) 4−18	7.1 (2.7) 4−12	8.5 (3.2) 4−14	8.4 (3.1) 4−14
Psychological barriers to care	Continuous Scale	12.8 (5.3) 7−30	12.6 (5.3) 7−30	12 (5.2) 7−30	13 (4.8) 7−26	14.3 (5.7) 7−26	12.9(4.6) 7−21
*Spouse Depression	Score < 5	221 (68%)	168 (69%)	52 (61%)	12 (55%)	24 (71%)	17 (68%)
	Score ≥ 5	102 (36%)	74 (31%)	34 (40%)	10 (46%)	10 (29%)	8 (32%)

*Note*: *NH* non-Hispanic, *MH* Mental Health, *Tx* Treatment

*A score of ≥ 5 is indicative of clinically significant depression symptoms

## Measures

### Mental Health Outcome

Depression, which is often comorbid with other measures of mental health such as anxiety, was used as a measure of general mental well-being [43]. Depression was measured by the Patient Health Questionnaire-8 (PHQ-8; [44]), on a 4-point scale, ranging from 1 (*not at all*) to 4 (*nearly every day*) and summed with higher scores indicating greater severity. Items include “little interest or pleasure in doing things” and “feeling tired or having little energy.” Internal consistency for the PHQ-8 was good in this sample (Cronbach’s alpha = 0.89) [44]. This scale was converted into a fuzzy set condition for configurational analysis. Sum scores were transformed into the log odds and sorted based on clinically relevant cut points so final analyses report on clinically significant depression (see supplemental materials for further detail about calibration process).

### Structural Determinants

Five demographic measures were included: (1) education (less than a bachelor’s degree/bachelor’s degree or more); (2) employment status (employed/unemployed); (3) race/ethnicity (non-Hispanic White/non-Hispanic Black/Hispanic/non-Hispanic other [Asian/Pacific Islander and those that marked “Other”]); (4) rank (enlisted/officer); (5) age (18–29 years/30 + years). History with the military was assessed with one item, which asked spouses to endorse three possible experiences: “I am/was a military service member,” “I grew up in a military family,” or “I was a military spouse in a prior marriage.” Any yes response was indicative of a personal history with the military.

### Social Cohesion and Capital Determinants

Three constructs were included in models: (1) social support, (2) sense of Army community, and (3) religious affiliation. Social support was assessed with three items from the Medical Outcomes Study (MOS) Social Support survey [45], which began with the prompt: “How often is each of the following kinds of support available to you if you need it?” Responses are on a 5-point Likert scale from 1 (*none of the time*) to 5 (*all of the time*); higher scores indicate greater social support. Examples of support include “Someone to give you good advice about a crisis,” and “Someone to take you to the doctor if you needed it.” Internal consistency was high in this sample (Cronbach’s alpha = 0.87) [45].

Sense of belonging to the Army community was assessed with a four-item scale developed by the Army [25]. Items included “I feel I am part of the Army community” and “I have friends from the Army community with whom I spend time socializing.” Responses are on a 5-point Likert scale from 1 (*strongly disagree*) to 5 (*strongly agree*); higher scores indicate greater sense of community. Internal consistency was good in this sample (Cronbach’s alpha = 0.81) [25]. Finally, spouses were asked, “Do you belong to a church, temple, or other religious group?” with two response options: 1 (*yes*)/0 (*no*).

### Intermediary Determinants

Nine constructs were included: distance to military installation, recent childbirth, recent injury or illness, family health/size, ACEs, IPV, work-family conflict, current mental health treatment received, and psychological barriers to mental health treatment. One question assessed residence on a military installation: “How far do you live from the nearest military installation (or the one you use the most)?” All responses other than I live on post (0) were recoded as Off post (1). Three respondents marked, “Do not know,” and were recoded as missing. Recent history of illness and/or childbirth was assessed with two items beginning with the prompt: “Within the past year, did any of following stressful events occur?” Items included “personal injury or illness” and “birth of a child” and included two response options: 1 (yes)/0 (no). Family health was assess through the perception of mental health treatment needs of the soldier through the question, “Have you noticed any behavior(s) in your spouse that makes you think they need mental health treatment?” 1 (yes)/0 (no). Number of children was measured through the question, “How many children to you have?” with a continuous response ranging from 0 to 7+. 

A modified version of the ACEs survey was employed [47–49], which assessed seven categories of lifetime childhood exposure to maltreatment and household dysfunction (psychological, physical, and sexual abuse, and household dysfunction, including substance abuse, mental illness, domestic violence, and incarceration) [30]. Responses were recoded to reflect any lifetime exposure (1) compared to none (0). IPV was assessed with a previously validated 10-item screener for clinically significant period prevalence of IPV, similar to the Physical Assault subscale of the revised Conflict Tactics Scales [50, 51]. Therefore, any “yes” response to the questions about physical or sexual abuse in the past year was considered clinically significant IPV.

Work-family conflict was assessed with the validated Work-Family Conflict five-item scale [52], modified to reference the service members’ job. Example items include “the demands of my spouse’s work interfere with my home and family life.” Responses were on a 7-point Likert scale from 1 (*strongly disagree*) to 7 (*strongly agree*). This scale’s internal consistency was strong in this sample (Cronbach’s alpha = 0.92) [52]. Mental health treatment of the spouse (non-military) was assessed through the question, “Are you currently in mental health treatment?” with response options: 1 (yes)/0 (no). Lastly, psychological barriers were measured with a seven-item scale, starting with the prompt: “Please rate how much you agree or disagree with the following factors related to receiving mental health counseling or services.” Responses are on a 5-point Likert scale from 1 (strongly disagree) to 5 (strongly agree). Examples include “It would be too embarrassing” and “I would be seen as weak.” This scale, originally validated for active duty service members, was adapted for this survey and demonstrated high internal consistency in this sample (Cronbach’s alpha = 0.91) [46].

## Analytic Plan

STATA 16.1 was used to assess descriptive statistics for each SDoMH category across each racial/ethnic identities and preliminary analysis to assess confounding variables. R Studio, R, and the R packages “cna”, “QCA”, and “SetMethods” were used for the CCMs analysis. This paper is one of the first to use coincidence analysis (CNA) as an exploratory method for factor selection prior to modeling with qualitative comparative analysis (QCA). All potential factors outlined in Fig. 1 will be considered for each racial/ethnic group. CNA is a relatively new member of the larger family of CCMs, featuring a unique bottom-up approach [53–56]. QCA has been employed in hundreds of studies dating back to the 1980s (see COMPASS.org for bibliography).

This study utilized fuzzy set QCA (fsQCA) so each condition or outcome was assigned a set membership value ranging from full-set membership (1) to full-set *non*membership (0), allowing for partial membership in one or more sets. For continuous factors, dual calibration was conducted in order to illustrate qualitative differences in the scale used [57] (Table 2). For example, the factor of social support was dual calibrated as “low social support” and “high social support.” The choice to use fsQCA was to best capture the continuous nature of the outcome of depression symptoms. For the present analysis, we used CNA to inform factor selection among the original 20 SDoMH factors and then fsQCA to model the complex pathways that produce and do not produce clinical depression symptoms (see supplemental materials for a complete description).

**Table 2:** Calibration of factors considered for final analysis

Construct	Fuzzy sets	Fully in	Crossover	Fully out
Personal History with Military	Yes, has a personal history with military	1	.49	0
**Rank**	Yes, is a specific rank (i.e., Officer, E1E4, E5E9)	1	.49	0
**Employment Status**	Yes, has a certain employment status (i.e., Employed, Unemployed not looked for work, Unemployed Looking for work)	1	.49	0
**Education**	Yes, has a bachelors +	1	.49	0
Age	Yes, 18−29 years old (young)	1	.49	0
**History of ACEs**	Yes, has a history of ACEs	1	.49	0
Distance to Military Installation	Lives on post	1	.49	0
Recent Childbirth	Yes, had a recent childbirth	1	.49	0
**History of Illness**	Yes, had a recent injury/illness	1	.49	0
Familial Health	Yes, thinks their spouse service member needs MH TX	1	.49	0
Family Size	Yes, has at least one child	1	.49	0
*Work-family Conflict (5-item scale with 7-point Likert response)	Low conflict: The fully in score was set at the equivalent of “disagree” of for the majority of items. The crossover was set between “neutral” and “agree.” The fully out score was set at the equivalent of “agree” on the majority of items.	18	19.1	25
	High conflict: The fully in score was set at the equivalent of “agree” for the majority of items. The crossover was set so the majority of the items were “neutral.” The fully out score was set at the equivalent of “disagree” on the majority of items.	22	18.1	17
Treatment Received	Yes, is receiving MH TX	1	.49	0
*Logistical Barriers to Care (4-item scale with 5-point Likert response)	Low Log. Barriers: The fully in score was set at the equivalent of “strongly disagree” for the majority of items. The crossover was set so the majority of the items were “neutral.” The fully out score was set at the equivalent of “agree” on the majority of items.	5	9.5	14
	High Log. Barriers: The fully in score was set at the of equivalent “agree” for the majority of items. The crossover was set so the majority of the items were “neutral.” The fully out score was set at the equivalent of “strongly disagree” on all items.	11	10.5	4
*Psychological Barriers to Care (7-item scale with 5-point Likert response)	Low Psych. Barriers: The fully in score is set at the equivalent of “disagree” on each item. The crossover was set between “neutral” and “agree.” The fully out score was set at the equivalent of “agree” on the majority of items.	14	16.9	23
	High Psych. Barriers: The fully in score was set at the equivalent of “agree” on each item. The crossover was set between “neutral” and “disagree.” The fully out score was set at the equivalent of “disagree” on the majority of items.	20	17.5	13
*Social Support (3-item scale with 5-point Likert response)	Low support: The fully in score was set at the equivalent of “None of the time,” and “a little of the time,” for the majority of items. The crossover was set between “a little of the time” and “some of the time.” The fully out score was set at the equivalent of “Most of the time,” and “All of the time” for the majority of items.	5	6.5	13
	High support: The fully in score was set at the equivalent of “Most of the time,” and “All of the time” for the majority of items. The crossover was set between “some of the time” and “most of the time.” The fully out score was set between “None of the time,” and “a little of the time” for the majority of items.	13	11.5	6
*Army Community (4-item scale with 4-point Likert response)	Low community: The fully in score was set at the equivalent “disagree/strongly disagree” for the majority of items. The crossover was set between “disagree” and “neutral.” The fully out score is set at the equivalent “agree” for the majority of items	7	11.1	15
	High community: The fully in score was set at the equivalent “agree” for the majority of items. The crossover was set between “neutral” and “agree.” The fully out score was set at the equivalent “disagree” for the majority of items	15	12.5	8
Religious Group Involvement	Yes, belongs to a religious group	1	.49	0
**Intimate Partner Violence** (IPV)	Yes, has clinically significant IPV	1	.49	0
*Mental Health Symptoms	Clinically Significant Depression Symptoms: The established clinical cut point of 5 and 10 for mild symptoms and a probable diagnosis of depression was used to inform fully in, crossover, and fully out cut points. Fully in was set at 9.9 so all scores of 10 or more would be considered fully in. Crossover was set to 4.9 so a score of 5 or more would be considered closer to fully in than out. Fully out was set to 4.1 so a score of 4 or less would be considered fully out.	9.9	4.9	4.1

*Indicates a fuzzy set calibration of a continuous scale and requires an explanation for fully in/out and crossover point selections. The dichotomous factors do not. The crossover point was always selected to err on the side of capturing the adjective in front of the factor name (i.e. high vs. low). See methods section for explanation of dual method calibration (i.e. having high vs low for one factor). Since we were only interested in low mental health symptoms we did not do a dual calibration for our outcome

*MH* Mental health, *TX* Treatment

**Bold** indicates the factor was used for final analysis

## Results

In preliminary analyses, pairwise correlations revealed variables are not multicollinear (not shown). Results from the exploratory CNA on each racial/ethnic subgroup identified different subsets of candidate factors to consider during the modeling phase with fsQCA. These candidate factors were represented in configurations with the strongest connections within each group to the outcome of clinically significant depression (see Table 3).

**Table 3:** Relevant conditions for clinically significant depression symptoms for Army wives

Condition(s)	Consistency	Coverage	Complexity
*Non-Hispanic Black*			
High psychological barriers to care	0.84	0.27	1
~ Bachelor’s degree * History of ACES	0.82	0.59	2
~ High social support * ~ Employed	0.81	0.50	2
High work-family conflict * Recent Illness/Injury	0.88	0.43	2
~ Officer * ~ High social support	0.81	0.37	2
~ Officer * ~ Bachelor’s degree * Clinically significant IPV	0.84	0.43	3
*Hispanic*			
E1E4 * ~ On post * Birth of recent child	0.78	0.54	3
~ High logistical barriers to care * ~ High social support * Clinically significant IPV	0.78	0.37	3
*Junior Enlisted non-Hispanic White*			
High work-family conflict * ~ On post * History of ACES * Low Army Community	0.76	0.26	4
~ Low work-family conflict * ~ On post * History of ACES * ~ High Army Community	0.76	0.26	4
*Non-Hispanic other*			
High work-family conflict * Recent injury/illness	0.87	0.39	2
Recent injury/illness * ~ High social support	0.82	0.31	2
Low Army community * ~ Military History	0.80	0.55	2
High work-family conflict * High logistical barriers * Low Army community	0.86	0.52	3

*Denotes the logical sign “AND” meaning both conditions must be present together

~  indicates the absence of the factor

Underlined condition denotes conditions included in the final QCA analysis. See the “Results” section for why other conditions were not included in the QCA analysis

Complexity means the number of conditions in the configuration. This table is a subset of the full CNA MSC results that met the consistency threshold of 0.8 and coverage threshold of 0.25 and thus were considered for the QCA analysis

Analysis did not result in a viable model for non-Hispanic White participants. This led to the analytic decision to examine a subgroup within this larger group that is typically not reported on and has been known to be at greater risk for major depressive disorder—junior ranking spouses [3]. Specifically, we examined junior enlisted spouses (*N* = 87 complete cases) which represented about a third of the overall total of non-Hispanic White respondents.

Each candidate factor was considered separately for fsQCA analysis. Necessity analysis on all the conditions and their negation for each subgroup, including items with specified directionality (i.e., high social support vs. low social support), revealed that only one met the 0.9 threshold recommended [39]. However, as has been done in other fsQCA work, it is acceptable to use the highest-scoring conditions that most aligned with a study’s conceptual framework [41]. The highest-scoring conditions that were aligned with the SDoMH for each racial/ethnic subgroup were *non-Hispanic Black*: high psychological barriers to care (0.27), employed (0.50), history of ACEs (0.69), high social support (0.45); *Hispanic*: E1E4 rank (0.54), living off post (0.97), recent childbirth (0.87); *junior enlisted non-Hispanic White*: high work-family conflict (0.75), living off post (0.68), high Army community (0.69), ACEs (0.77); *non-Hispanic other race*: high work-family conflict (0.83), low Army community (0.55), not having a recent injury/illness (0.63), not having a military history (0.80).

Sufficiency analysis revealed different pathways for each racial/ethnic group for clinically significant depression symptoms. Consistent with best practices, we also report the characteristics of each truth table, including the number of rows and cases with consistency values of 0.75 or higher and the number of rows with no cases (see supplementary materials). Table 4 shows the results of the fuzzy set analysis of clinically significant depression, and Table 5 shows the negative model for not having clinically significant depression.

**Table 4:** Pathways for *presence of* clinically significant depression symptoms by race/ethnicity

		non-Hispanic Black		Hispanic	Junior Enlisted non-Hispanic White	non-Hispanic Other
Domain	Explanatory Conditions	PATH1	PATH2			
*Structural Determinants*	Employed	●	○			
	E1E4			●		
	Military History					○
*Social Support & Social Cohesion*	Social Support (High)		○			
	Army Comm. (High)				○	
	Army Comm. (Low)					●
*Intermediary Determinant*	History of ACEs	●	●		●	
	Psychological Barriers to Mental Health Care (High)	●	○			
	Recent Childbirth			●		
	Live on Post			○	○	
	Work-Family Conflict (High)				●	●
	Recent Injury/Illness					
Consistency		.88	.92	.78	.76	.87
Raw coverage^a^		.24	.40	.54	.26	.53
Unique coverage^b^		.20	.36	.54	.26	.53
Overall solution consistency		.92		.78	.76	.87
Overall solution coverage^c^		.60		.54	.26	.53

● indicates the presence of the condition, ○ indicates the absence of the condition

^a^Indicates how much of the outcome (clinically significant depression symptoms) is covered by the solution

^b^Indicates how much of the outcome (clinically significant depression symptoms) is uniquely covered by the solution

^c^Indicates how much of the outcome (clinically significant depression symptoms) is covered by all solutions taken together

**Table 5:** Pathways for *absence of* clinically significant depression symptoms by race/ethnicity

		non-Hispanic Black		Hispanic			Junior Enlisted non-Hispanic White			non-Hispanic Other
Domain	Explanatory Conditions	PATH1	PATH2	PATH1	PATH2	PATH3	PATH1	PATH2	PATH3	
*Structural Determinants*	Employed	●	○							
	E1E4			○						
	Military History									
*Social Support & Social Cohesion*	Social Support (High)	●	●							
	Army Comm. (High)								●	
	Army Comm. (low)									○
*Intermediary Determinant*	History of ACEs	●	○				○	○		
	Psychological Barriers to Mental Health Care (High)	○	○							
	Recent Childbirth					○				
	Live on Post				●		○	○	●	
	Work-Family Conflict (High)						○			
	Recent Injury/Illness									○
Consistency		.91	.92	.84	.99	.92	.86	.80	.81	.89
Raw coverage^a^		.27	.26	.61	.99	.91	.31	.31	.39	.88
Unique coverage^b^		.20	.20	.23	.56	.38	.16	.14	.32	.88
Overall solution consistency		.94		.89				.81		.89
Overall solution coverage^c^		.46		.88				.79		.88

● indicates the presence of the condition, ○ indicates the absence of the condition

^a^Indicates how much of the outcome (absence of clinically significant depression symptoms) is covered by the solution

^b^Indicates how much of the outcome (absence of clinically significant depression symptoms) is uniquely covered by the solution

^c^Indicates how much of the outcome is covered by all solutions taken together

### Pathways for the Presence of Clinically Significant Depression Symptoms

All solutions demonstrated good consistency (> 0.75) and material coverage (> 0.25). The junior enlisted non-Hispanic White group had the lowest coverage score of 0.26, which is acceptable [39]. The best fitting solution was for non-Hispanic Black Army wives (consistency: 0.92, coverage: 0.60). Results show that each race/ethnicity had different pathways of conditions that adversely affected depression symptoms (see Table 4).

### Pathways for the Absence of Clinically Significant Depression Symptoms

All solutions for the absence of the outcome demonstrated strong consistency (> 0.81) and adequate coverage (> 0.46). The highest consistency score was for non-Hispanic Black participants (0.94), and the highest coverage score was 0.88 for both Hispanic and non-Hispanic other race participants. Results show that each race/ethnicity has different pathways which explain the absence of clinically significant depression (see Table 5).

No PRI scores were below 0.6, indicating that there were no significant inconsistencies with the pathways found [58].

## Discussion

This paper adapted the WHO SDoMH conceptual framework to the military context to conceptualize how structural, social cohesion and capital, and intermediary determinants interact to affect the mental health of Army wives. The paper employed CCMs to identify eight different solutions that accounted for clinically significant depression symptoms among Army wives across four racial/ethnic groups. Four models consistently explained the presence of clinically significant depression symptoms and four models consistently explained the absence of clinically significant depression symptoms; models differed across racial/ethnic minority groups. Findings highlight how different determinant conditions combine to lead to clinically significant depression symptoms across racial and ethnic groups of Army wives.

Solutions leading to clinically significant depression—and its absence—demonstrate this complex outcome is predicated on a combination of conditions for each racial/ethnic group, rather than on a single determinant. Conditions that were relevant across groups included a history of ACEs, absence of high social support, living off post, and high-work-family conflict, consistently representing two of the SDoMH determinant groups. In addition to military-specific stressors, Army wives experience potential stressors in their everyday lives including ACEs, low social support, living away from a military installation, and high levels of work-family conflict. These potential stressors, not specific to their military connection, appear to be important in understanding risk for adverse mental health outcomes in this population [5,59].

These findings also suggest that Army wives are not a monolithic group. Across racial/ethnic subgroups, conditions combine in ways that create different pathways to poor mental health. For example, though ACE was a common condition across groups, this determinant combined with *different *conditions to explain clinically significant depression. For non-Hispanic Black Army wives, these contingent conditions included being employed and experiencing high psychological barriers to mental healthcare, while for junior enlisted non-Hispanic White Army wives, they included a lower sense of Army community, living off post, and high work-family conflict. These findings highlight a need for holistic mental health assessments to explore unique SDoMH factors that could be impacting mental well-being. For example, clinicians serving this population might consider a biopsychosocial approach, which incorporates an individual’s biological, psychological, and social history to inform the best course of treatment [60, 61].

Findings also emphasize the importance of social support. For several racial/ethnic groups, ACEs led to depression only in the absence of social support. This finding is consistent with the SDoMH framework which conceptualizes social support as intersecting structural and intermediary determinants, suggesting this factor can interrupt a potential negative health trajectory [7]. This finding is also in line with empirical evidence suggesting social support may buffer the effects of adverse childhood events on later life depression symptoms [62]. The replication of this finding in a military spouse population is useful as providers could support wives with a history of ACEs by increasing their supportive social connections.

These analyses produced different findings regarding living on/off post for different racial/ethnic groups. Depending upon the specific model, either living on or off post was linked to clinically significant depression. These findings may be understood in the context of spouse’s views toward the military. For example, living on post could be a positive experience for wives when it facilitates access to resources like medical services and connections to other military families. However, living on post could be a negative experience for spouses who hold negative views of the military and value distance from military culture [1]. Living on post can also isolate military wives from civilian friends and family members [63]. Living on post in the presence of a high sense of Army community, potentially associated with positive views of the military, led to positive mental health outcomes for junior enlisted non-Hispanic Army wives.

While there were some similarities across models, no two racial or ethnic groups were the same; within each group, there were different crucial determinants. For example, combinations of employment and social support were important in explaining depression for non-Hispanic Black Army wives. Employment in the absence of social support linked directly to clinically significant depression, whereas employment in the presence of social support linked directly to low or no depression symptoms. This finding supports recent research that Black women in the workplace have to consistently balance fitting into their workplace environment with their own uniqueness which can affect their ability to feel supported and included [64, 65]. For Hispanic Army wives, having a recent childbirth (within the past year) combined with living off post and being partnered with a junior enlisted soldier connected directly to clinically significant depression symptoms. This could be for several reasons, including lower income levels that junior enlisted service members receive which could create family financial strain leading to mental distress [66]. Another reason could be the prevalence of non-traditional medicine beliefs among Hispanic parents and how these beliefs can conflict with western medicine practices [67]. A recent birth for a Hispanic mother may be particularly stressful, given that she may have to balance health information from her culture and family (which she could have more access to living off post) with that of her practitioner. These results highlight that even in a population with access to universal healthcare, there are racial/ethnic differences in life stressors as well as opinions of receiving mental healthcare in general (i.e., psychological barriers to care) that contribute to clinically significant depression.

These findings support the use of the adapted SDoMH framework in understanding the crucial set of factors that can impact military spouses’ mental health across racial/ethnic groups. Further, this study is methodologically innovative in its pairing of CNA with fsQCA to systematically consider combinations of potentially relevant determinants across groups. Using fsQCA, this study illustrated the interconnectedness among conditions that may affect clinically significant depression symptoms for racially and ethnically diverse Army wives. Fuzzy set QCA has demonstrated promising applications to understanding racial disparities [41,57]. For example, rather than attributing differences in poverty across racial and ethnic groups to test scores in school, Ragin and Fiss [57] used fsQCA to show how accumulated advantage favors White students and accumulated disadvantage disfavors Black students. Rich and colleagues [41] used fsQCA to combine qualitative data with quantitative measures and found combinations of levels of trauma symptoms, financial worry, and discrimination were sufficient to explain help-seeking behavior among Black male trauma survivors. Applying this approach in health research can help account for complex outcomes related to health inequalities rather than focusing on specific demographic categories such as race or gender that are often investigated in isolation of their socio-ecological context [41, 68].

### Limitations

Findings should be interpreted in light of several limitations. The present study was a secondary analysis; therefore, several potentially relevant factors were not available for inclusion in models, such as the use of anti-depressant medications, lifetime prevalence of IPV, substance use, racial stress, or discrimination. Instead, this study used proxies of some of the structure determinants outlined in the WHO framework to assess their impact on depression. This study’s generalizability is limited because a non-probability sampling approach was used with a 23% response rate from eligible spouses. Analyses are based on self-report data which could introduce common method bias. For example, spouses with poor mental health may be more likely to report low social support. Furthermore, as this is an observational study, determining the strength and direction of any causal relationships would require additional evidence like RCTs and independent replication of results.

It is important to note that our findings identify SDoMH factors linked to depression among subgroups in our dataset but may not generalize to larger populations. Specifically, our subsamples are small, have different distributions of the SDoMH factors between race/ethnicities, and may not be nationally representative of racial/ethnic subgroups of military wives. However, at this time, there are no national data available on the demographic differences assessed in this study between racial/ethnic subgroups of military spouses, making it difficult to assess how representative our sample is of the larger Army/military spousal community.

Calibration is a critical step in the fsQCA process that is described in detail in the supplemental materials and shown in Table 2, and results are sensitive to different calibrations of the explanatory conditions and outcomes. We provided details about how we arrived at these calibrations. Nonetheless, other researchers might construct calibrations with different set membership parameters. To address this concern, we used the skew.check function when creating the calibration cut points of fully in, crossover, and fully out. This function allowed us to test how different cut points could skew the data ± 10% to ensure we were accurately capturing the data. The results showed that even if the cut points for calibration varied by ± 10%, the distribution of the data did not significantly change, supporting our cut point decisions.

For non-Hispanic White Army wives, we were not able to model depression, likely due to heterogeneity in this group, which impeded our ability to identify a path of conditions leading to depression [69]. Additionally, while this study tried to examine the diversity of Army wives racial/ethnic identities, certain decisions were made to ensure adequate numbers for the analysis. This included the creation of the “Other” racial/ethnic category that included Asian and Pacific Islanders as well as individuals who responded “Other.” Future research with larger sample sizes should separate these racial/ethnic groups as there is likely more heterogeneity that could be assessed.

### Implications

Though preliminary, this paper identified complex pathways for clinically significant depression symptoms in Army wives. Rather than one determining factor, this study highlighted a combination of conditions which interact to affect depression symptoms differently within each racial/ethnic group. Findings highlight how Army wives are not a monolithic group, despite their collective exposure to military-specific stressors.

Collectively, these findings suggest that mental health assessments should take into account spouses’ larger contexts in order to fully understand conditions that could impact their mental health. Future research using the SDoMH model and CCMs should incorporate qualitative data to examine in greater depth and detail how qualitative differences in environmental and life factors affect mental health.

### Supplementary Information

Below is the link to the electronic supplementary material.

Supplementary file1 (DOCX 39.4 KB)

## Data Availability

Data is not available for public access because institutional policies require that data use agreements have to be in place before data can be shared.

## Code Availability

The code, excluding specific variables used (to ensure patient safety and confidentiality), can be shared upon request of corresponding author, Dr. Dodge, jessica.dodge@va.gov.
