# Supplementary material for: Exploring the Social Determinants of Mental Health by Race and Ethnicity in Army Wives
Source: J Racial Ethn Health Disparities. 2023 Mar 23;11(2):669–84. doi: 10.1007/s40615-023-01551-3 (PMC10933139; doi:10.1007/s40615-023-01551-3)
Supplement: Supplementary file 1 — Supplementary file1 (DOCX 39.4 KB) [file 40615_2023_1551_MOESM1_ESM.docx]

**Supplemental Materials**

**Step by Step Analytic Plan**

***CCMs-Step One***

The first step in the fsQCA analysis is calibration—the process of assigning membership scores to cases based on empirical and theoretical knowledge ^1^. For dichotomous factors, we conducted direct method calibration ^1,2^. With direct method calibration, cut points (fully in [.95], crossover [.5] , fully out [.05]) are established through theory, substantive knowledge, and prior empirical work ^1,2^. We first determined the value of the interval scale of measurement score at which a case is “fully in” a specified set (i.e., set membership score .95); the crossover point, or threshold for maximum ambiguity or at which point the case membership score is .5 in the set and .5 out of the set; and finally, the value at which a case is “fully out” of a set (i.e., set membership score of .05). We then transformed the original interval scale values into fuzzy set membership scores by transforming these data based on the log odds of full membership, using the “calibrate” command in the QCA package in R.

For continuous factors, dual calibration was conducted in order to capture the variation in scores that does not exist with dichotomous factors. In dual method calibration, the direct method is extended even further so a single factor is categorized into more than one condition to illustrate qualitative differences in the scale used ^2^. For example, the variable of social support is divided up into conditions “low social support” and “high social support,” with appropriate cut-points of fully in, crossover, and fully out. For a full list and description of the 20 factors tested in the factor selections process, as well as their calibration, see Tables 1 & 2.

***CCMs-Step Two***

The second step utilized a bottom-up approach for factor selection. This bottom-up approach operates within the same regularity framework as fsQCA and has been used in other peer-reviewed CCMs-related work within health care ^3–8^. The bottom-up approach of CNA is well suited to inform factor selection by exhaustively searching every combination of conditions instantiated in the entire dataset and identifying minimally sufficient conditions that meet selection criteria. Minimally sufficient conditions can be thought of as an individual or a collection of calibrated conditions within the data that meet a preset consistency threshold.

Consistency and coverage are two parameters often used to measure the strength of set relationships, and both were used as key specifications in this data reduction phase of the analysis ^3^. For crisp-set QCA, consistency is the number of cases that have the outcome present and are covered by a given configurational solution divided by all cases covered by that given configurational solution. Coverage is the number of cases that have the outcome present and are covered by a given configurational solution divided by all cases with the outcome. Consistency and coverage in fuzzy-set QCA has the same conceptual basis but is calculated somewhat differently (see Schneider & Wagemann, 2012).

For a condition to be included in the final fsQCA analyses, we used the “msc” function in the R package “cna” to look across all 20 factors within each racial/ethnic subsample to identify configurations with the strongest apparent connections to clinically significant depression symptoms. The strongest condition(s) were determined by the following criteria as outlined by Yakovchenko et al. (2020): (1) configuration met the consistency threshold of .75; (2) coverage score for configuration was at least .25 and was uniquely distinguishable from all other configurations sharing the same complexity level (to guard against overfitting); (3) configuration aligned theoretically with the adapted WHO SDoMH framework and prior published work about military spouse mental health.

***CCMs-Step Three***

After factor selection, standard fsQCA procedure was used ^1^. Necessity and sufficiency analysis were conducted on relevant factors from the mscs factor selection process to produce pathways that produce our outcome of interest—clinically significant depression symptoms across racial/ethnic subsamples of Army wives. Necessity analyses identify any causal conditions that must be present for the outcome to occur. Consistent with other fsQCA literature, we used the consistency level of .9 or above as the threshold for determining if a causal condition was necessary ^1^. Sufficiency analyses help identify which causal condition or combination of conditions consistently produce an outcome.

Sufficiency analyses primarily rely on truth tables that display all logically possible combinations of causal conditions. For this study’s outcome of clinically significant depression symptoms, a consistency threshold of .8 was used which is consistent with other fsQCA literature ^1^. Standard analysis using the Quine-McCluskey algorithm was applied to the truth table to find the pathways that consistently lead to clinically significant depression for the majority of each racial/ethnic subsample of Army wives. Consistent with other fsQCA literature, this analysis utilized the pathways generated by the intermediate solution ^1,9^.

Since fsQCA does not assume the opposite solution for the absence of the outcome (in this case absence of clinical depression symptoms), it is recommended that the negation of the outcome be analyzed to explore any further explanations of causal conditions that may be different than configurations that explain the presence of clinically significant depression symptoms ^1^. Lastly, the Proportional Reduction Inconsistency (PRI) scores were checked to avoid relations of configurations in both the outcome and its absence, which would not make logical or conceptual sense ^10^.

**Supplemental Table 1**. Truth Table Characteristics.

|  | **Rows (cases) with Consistency ≥ 0.80** | **Rows (cases) with Consistency ≤ 0.80** | **Row with No Cases (Logical Remainders)** |
| --- | --- | --- | --- |
| Non-Hispanic Black | | | |
| Clinically Significant Depression | 3 (8) | 5 (8) | 0 |
| *Absence of* Clinically Significant Depression | 2 (8) | 6 (8) | 0 |
| Hispanic | | | |
| Clinically Significant Depression | 1(8) | 7(8) | 0 |
| *Not* Clinically Significant Depression | 7(8) | 1(8) | 0 |
| Junior Enlisted non-Hispanic White | | | |
| Clinically Significant Depression | 1 (14) | 13 (14) | 0 |
| *Not* Clinically Significant Depression | 10 (14) | 4 (14) | 0 |
| Non-Hispanic Other | | | |
| Clinically Significant Depression | 2(7) | 5(7) | 0 |
| *Not* Clinically Significant Depression | 4(7) | 3(7) | 0 |

Logical remainders= rows in the truth table without any cases

| **Determinant Category** | **Construct** | **Level of Measurement** | **Operationalization & Variable Description** |
| --- | --- | --- | --- |
| *Structural Determinant* | Personal history with military | Dichotomous | (Yes) to any of the following: grew up in military family, prior military spouse, prior personal military history vs. (No) |
|  | Race/ethnicity | Categorical | White, Black, Hispanic, Other |
|  | Sex | *NA* | *All female sample* |
|  | Rank | Categorical | E1-E4, E5-E9, Officer/Warrant Officer |
|  | Employment status | Categorical | Employed full or part time, unemployed and seeking work, unemployed not seeking work |
|  | Education | Dichotomous | Bachelors + vs. < Bachelors |
|  | Age | Dichotomous | 30 + years vs. 18-29 years |
| *Social Capital & Social Cohesion* | Social support | 5-point Likert Response | **3-item scale**; e.g. People sometimes look to others for companionship, assistance, or other types of support. How often is each of the following kinds of support available to you if you need it? |
|  | Army community | 5-point Likert Response | **4-item scale**; e.g. Please rate how much you agree or disagree with the following statements. Examples items include, “"I feel I am part of the Army community |
|  | Religious group involvement | Dichotomous | Do you belong to a church, temple, or other religious group (yes/no) |
| *Intermediary Determinant* | Distance to military installation | Dichotomous | I live on-post vs. Not |
|  | Recent childbirth | Dichotomous | During the past year, did any of the following stressful events occur? Birth of child (yes/no) |
|  | History of illness | Dichotomous | During the past year, did any of the following stressful events occur? Personal injury or illness (yes/no) |
|  | Familial health | Dichotomous | Did you spouse have a serious illness while they were deployed? (no, yes most recent deployment, yes earlier deployment, yes most recent and earlier deployment) |
|  | Family size | Dichotomous | No children vs. 1+ |
|  | History of ACEs | Dichotomous | Yes exposure vs. No exposure |
|  | Intimate Partner Violence (IPV) | Dichotomous | Yes vs. No to clinically significant IPV |
|  | Work-family conflict | 7-point Likert Response | **5-item scale**: e.g. Please rate how much you agree or disagree with the following statements. Example items include, “the demands of my spouse’s work interfere with my home and family life.” |
|  | Treatment received | Dichotomous | Are you currently in mental health treatment? (yes/no) |
|  | Logistical barriers to care | 5-point Likert Response | **4-item scale**; How much to do you agree or disagree with the following factors related to receiving mental health counseling or services. |
|  | Psychological barriers to care | 5-point Likert Response | **7-item scale**; e.g. How much to do you agree or disagree with the following factors related to receiving mental health counseling or services. |
| *Outcome* | Depression symptoms | 4-point Likert Response | **8-item scale**; e.g. Over the past month how often have you been bothered by any of the following problems? |

**Supplemental Table 2.** All Measures Considered for Final Analysis.

**References**

1. Schneider CQ, Wagemann C. *Set-Theoretic Methods for the Social Sciences: A Guide to Qualitative Comparative Analysis*. (Elman C, Gerring J, Mohoney J, eds.). Cambridge: Cambridge University Press; 2012.

2. Ragin C, Fiss P. *Intersectional Inequality : Race, Class, Test Scores, and Poverty*. University of Chicago Press; 2017.

3. Yakovchenko V, Miech EJ, Chinman MJ, et al. Strategy configurations directly linked to higher Hepatitis C virus treatment starts: An applied use of Configurational Comparative Methods. *Med Care*. 2020;58(5):e31-e38. doi:10.1097/MLR.0000000000001319

4. Hickman SE, Miech EJ, Stump TE, Fowler NR, Unroe KT. Identifying the implementation conditions associated with positive outcomes in a successful nursing facility demonstration project. Meeks S, ed. *Gerontologist*. 2020;60(8):1566-1574. doi:10.1093/geront/gnaa041

5. Whitaker RG, Sperber N, Baumgartner M, et al. Coincidence Analysis: a new method for causal inference in implementation science. *Implement Sci*. 2020;15(1). doi:10.1186/s13012-020-01070-3

6. Knott CL, Miech EJ, Slade J, Woodard N, Robinson-Shaneman B-J, Huq M. Evaluation of Organizational Capacity in the Implementation of a Church-Based Cancer Education Program. *Glob Implement Res Appl 2022*. January 2022:1-12. doi:10.1007/S43477-021-00033-0

7. Miech EJ, Freitag MB, Evans RR, et al. Facility-level conditions leading to higher reach: a configurational analysis of national VA weight management programming. *BMC Health Serv Res*. 2021;21(1):1-9. doi:10.1186/S12913-021-06774-W/FIGURES/1

8. Cohen DJ, Sweeney SM, Miller WL, et al. Improving Smoking and Blood Pressure Outcomes: The Interplay Between Operational Changes and Local Context. *Ann Fam Med*. 2021;19(3):240-248. doi:10.1370/AFM.2668

9. Rich JA, Corbin TJ, Jacoby SF, Webster JL, Richmond TS. Pathways to Help‐Seeking Among Black Male Trauma Survivors: A Fuzzy Set Qualitative Comparative Analysis. *J Trauma Stress*. 2020;33(4):528-540. doi:10.1002/jts.22517

10. Greckhamer T, Furnari S, Fiss PC, Aguilera R V. Studying configurations with qualitative comparative analysis: Best practices in strategy and organization research. *Strateg Organ*. 2018;16(4):482-495. doi:10.1177/1476127018786487
